# The CD4+ T cell regulatory network mediates inflammatory responses during acute hyperinsulinemia: a simulation study

**DOI:** 10.1186/s12918-017-0436-y

**Published:** 2017-06-26

**Authors:** Mariana E. Martinez-Sanchez, Marcia Hiriart, Elena R. Alvarez-Buylla

**Affiliations:** 1 Genética Molecular, Desarrollo y Evolución de Plantas, Departamento de Ecología Funcional, Instituto de Ecología, Universidad Nacional Autónoma de México, México, Mexico; 2 Centro de Ciencias de la Complejidad, Universidad Nacional Autónoma de México, México, Mexico; 3 Departamento de Neurociencia Cognitiva, Instituto de Fisiología Celular, Universidad Nacional Autónoma de México, México, Mexico

**Keywords:** CD4+ T cells, Hyperinsulinemia, Regulatory network, Plasticity, Robustness, Development, Insulin, IL-10, TGFβ

## Abstract

**Background:**

Obesity is frequently linked to insulin resistance, high insulin levels, chronic inflammation, and alterations in the behaviour of CD4+ T cells. Despite the biomedical importance of this condition, the system-level mechanisms that alter CD4+ T cell differentiation and plasticity are not well understood.

**Results:**

We model how hyperinsulinemia alters the dynamics of the CD4+ T regulatory network, and this, in turn, modulates cell differentiation and plasticity. Different polarizing microenvironments are simulated under basal and high levels of insulin to assess impacts on cell-fate attainment and robustness in response to transient perturbations. In the presence of high levels of insulin Th1 and Th17 become more stable to transient perturbations, and their basin sizes are augmented, Tr1 cells become less stable or disappear, while TGFβ producing cells remain unaltered. Hence, the model provides a dynamic system-level framework and explanation to further understand the documented and apparently paradoxical role of TGFβ in both inflammation and regulation of immune responses, as well as the emergence of the adipose Treg phenotype. Furthermore, our simulations provide new predictions on the impact of the microenvironment in the coexistence of the different cell types, suggesting that in pro-Th1, pro-Th2 and pro-Th17 environments effector and regulatory cells can coexist, but that high levels of insulin severely diminish regulatory cells, especially in a pro-Th17 environment.

**Conclusions:**

This work provides a first step towards a system-level formal and dynamic framework to integrate further experimental data in the study of complex inflammatory diseases.

**Electronic supplementary material:**

The online version of this article (doi:10.1186/s12918-017-0436-y) contains supplementary material, which is available to authorized users.

## Background

Obesity-associated chronic inflammation is a complex phenomenon that results from the interaction between adipose tissue, hyperinsulinemia, and chronic inflammation [1–4]. Together, these linked conditions increase the risk to develop metabolic syndrome and type-2 diabetes mellitus. To understand how such complex syndrome emerges, it is necessary to use an integrative, system-level and dynamic approach that takes into consideration: the non-linearity of the interactions, the strong effect of the environment, the constant crosstalk and feed forward or feed-back interactions among the genetic and non-genetic components involved, and the synchronic or concerted nature of various regulatory events and conditions involved [5–9]. Most studies have focused on the direct relationship between macrophages and obesity [10], meanwhile, important questions concerning the relationship between obesity, insulin, and CD4+ T cell type populations and plastic changes among them remain unaddressed. These probably play important roles in the onset of inflammatory responses, and their systemic impact remains unresolved. A starting point, involves understanding: (i) the complex regulatory network involved in the cell fate attainment of CD4+ T cell types [11–13], (ii) how such network responds to extracellular metabolic and micro-environmental conditions [14, 15], and (iii) how the resulting system dynamically modulates the inflammatory and immune responses [1–4].

Obesity-associated chronic inflammation results from prolonged excessive nutrient intake [16, 17]. Under such condition, adipocytes in the visceral adipose tissue (VAT) stimulate the inflammatory response by producing pro-inflammatory cytokines, increasing activated macrophages, but also altering the CD4+ T cell population which likely feedbacks to inflammation [16, 18]. This inflammatory response affects insulin signalling in the cells, promoting insulin resistance, as well as glucose metabolism, and together may indirectly promote an increase in insulin production by pancreatic beta cells [1–4, 16, 17, 19]. Hyperinsulinemia is strongly associated with metabolic syndrome that is a set of signs that increases the risk to develop type-2 diabetes mellitus, cardiovascular diseases and certain types of cancer. Metabolic syndrome is characterized mainly by: central obesity, hypertension, dyslipidemia, hyperinsulinemia, insulin resistance and glucose intolerance. Despite the fact that such syndrome is well described, the system-level underlying mechanisms, as well as the global health consequences associated with hyperinsulinemia have not been fully understood [19].

CD4+ T cells are fundamental modulators of immune challenges and the homeostasis of the immune system. Naive CD4+ T cells (Th0) are activated when they recognize an antigen in a secondary lymphoid organ. CD4+ T cells may attain different cell fates depending on the cytokine milieu, TCR stimulation, and other signals in their microenvironment [20, 21]. The cytokines can be produced by the lymphocyte (intrinsic) or by other immune cells (extrinsic). The different cell types express characteristic transcription factors and cytokines and have been associated with specific roles in the immune system [20]. The classification of CD4+ T cells in subsets has been complicated, as they are highly heterogeneous and plastic. There are reports of hybrid cells that express transcription factors and cytokines from more than one cell type [22, 23]. This is the case, for example, of T-bet + Foxp3 and GATA3 + Foxp3 cells [24, 25]. Furthermore, CD4+ T cells can plastically alter their expression patterns in response to environmental conditions [26–29]. Such complex and dynamic plastic behaviors have started to be explained at the system level using multi-stable network models [11–13].

Regulatory T cells maintain immune tolerance; regulate lymphocyte homeostasis, activation, and function. Regulatory T cells can be classified into various types. Treg cells are characterized by the transcriptional factor Foxp3, high expression of CD25+, and they produce TGFβ and IL-10. But, these two cytokines can also be expressed independently of Foxp3. TGFβ is necessary for the differentiation of regulatory Tregs and effector Th17 cells. TGFβ has a context-specific role in the immune response; it can suppress or enhance the immune reaction, depending on its cofactors [30, 32]. IL-10 is an immunosuppressive cytokine produced by many cells during an immune response, including Tr1 cells. It acts as a feedback regulator of the immune response by inhibiting the production of inflammatory cytokines [33]. Moreover, T cells that express T-bet or GATA-3, in addition to certain regulatory factors, are important in regulating the Th1 and Th2 response [24, 34, 35].

CD4+ T cells are involved in the inflammatory feedback loop in obesity-associated tissue inflammation. In obese VAT murine models and humans, an enrichment of the Th1 and Th17 populations and a decrease in regulatory T cells has been described [3, 36–38]. Th1 and Th17 cells produce proinflammatory cytokines that inhibit insulin signalling. The transcriptional profiles and functions of Tregs are also altered; by expressing proinflammatory cytokines like IFNγ and IL-17. This change in expression patterns causes Tregs to cluster with inflammatory T cells [3, 36–38]. While TGFβ is present in adipose tissue, its role in regulating Treg cells is still unclear, because the typical alterations caused by TGFβ are not observed in these cells [39]. Paradoxically, it has been reported that the reduction of adipose tissue Treg cells seems to both, improve and worsen insulin resistance [37–40]. This kind of behavior could be linked to an underlying multi-stable dynamic mechanism such as that recently proposed to study CD4+ T cell differentiation and plasticity [11–13]. On the other hand, the general metabolic state of an individual also affects CD4+ T cells. Obesity is associated with increased insulin levels, which affects CD4+ T cells. Insulin is necessary for the survival and proliferation of activated CD4+ T cells. Effector T cells, such as Th1, Th2 and Th17, depend on glycolysis, while resting (not activated), regulatory and memory T cells depend mainly on lipid oxidation. But in obese VAT, the high levels of insulin over-activate the AKT pathway, inhibiting IL-10 production and its regulatory functions in CD4+ T cells [15]. Hence, the relationship between insulin resistance and CD4+ T cells is still unclear.

We propose here a theoretical simulation study, to explore the molecular interactions between the previously published CD4+ T cell regulatory network [13] and hyperinsulinemia [15]. Such a system-level mechanistic approach is fundamental for understanding CD4+ T cell differentiation and plasticity dynamics at the cellular level in response to the metabolic state caused by hyperinsulinemic condition. We used the Boolean regulatory network for studying CD4+ T cell differentiation and plasticity dynamics in response to insulin. Boolean networks, although qualitative, can capture key aspects of complex biological behaviors without having detailed kinetic and temporal parameters. Hence, they are useful to study their dynamics under various stimuli and conditions [41]. The system studied here includes transcription factors, signalling pathways, intrinsic and extrinsic cytokines [13]. We use such system to evaluate the impact of basal and high levels of insulin [15]. The model recovers the differentiation of CD4+ T cells, including effector (Th1, Th2, Th17) and regulatory (iTreg, Th1R, Th2R, Tr1 and Foxp3-TGFβ + cells) cell types [13, 20]. Here, we show how hyperinsulinemia shapes CD4+ T cell attainment by reducing the production of IL-10 and causing a shift towards pro-inflammatory, resting, or TGFβ + producing cell types. Constant pro-regulatory signals can counteract this change. We also explore how the presence of high levels of insulin in the environment alters the plasticity of CD4+ T cell in response to transient fluctuations in the elements of the network. High insulin also favors transitions towards inflammatory, resting or TGFβ + producing cell types and reduces the stability of regulatory cell types. In this way, we show how a modified version of the CD4+ T cell molecular network model proposed before [13], that now includes IL12, IL-27, and hyperinsulinemia, seems to mediate the observed cellular behavior in obesity-associated chronic inflammation. This network model constitutes a useful framework to further explore the system-level mechanisms involved in inflammatory conditions including obesity.

## Methods

### Logical modelling formalism: Boolean networks

A Boolean network is composed of nodes that represent the system’s molecular components (i.e., cytokines, signalling pathways or transcription factors) and edges that represent the interactions between nodes. In any network model, the value of the nodes can be associated with a discrete variable denoting its current functional level of activity: In the Boolean formalism if the node is functional its value is 1, and if it is not functional it is 0. The value of a node *i* at the time *t + 1* depends on the value of all its input nodes or regulators at time *t*, according to the logical function of the target node as a function of its inputs [Additional file 1: Table S1]. For the Boolean case, the function of each network component or node is of the form:

$$ {x}_i\left( t+ 1\right)={f}_i\left({x}_1(t),{x}_2(t),\dots, {x}_k(t)\right) $$

where *x*_*i*_ is the value of the node *i*, *t* is the time, *f*_*i*_ is the Boolean function of the node *i*, and *x*_*1*_ *, …, x*_*k*_ are the values of its k regulators.

#### Model construction and reduction

For the construction of the network, the Boolean functions were defined based on available CD4+ T differentiation models [11–13] and experimental data for the reported interactions among a network of more than 90 nodes [Additional file 1: Table S2]. A transcription factor regulates another factor if it binds to the regulatory region of the latter factor and inhibits or activates its transcription. A cytokine is present if it is either secreted by the cell (intrinsic) or produced by other cells of the immune system (extrinsic). To separate the effects of the cytokines produced by the immune system from those of the cytokines produced by the CD4+ T cell, we label extrinsic cytokines as ILe. Receptors are considered to be active when the cytokine is stably bound to a receptor, enabling it to transduce a signal. STAT proteins are considered active when they are phosphorylated and capable of translocating to the nucleus. The activation of a STAT protein depends on the presence of interleukin, its correct binding to the receptor, and subsequent phosphorylation. SOCS proteins inhibit the phosphorylation of STAT by competing for the phosphorylation site. A protein or gene may be expressed at a basal level, but does not necessarily affect the differentiation of the cell at that level of expression, in this case we considered that the basal level of the protein corresponded to zero, while the higher level corresponded to one. The network was then simplified [Additional file 2: File S2] [13, 42, 43]. The resulting network has 19 nodes and 54 interactions. As part of the simplification we assumed that the signal produced by the TCR and its co-factors was active and enough to induce activation and ignored weak interactions as well as input and output nodes.

### Dynamic analysis

**Fig. 1:**
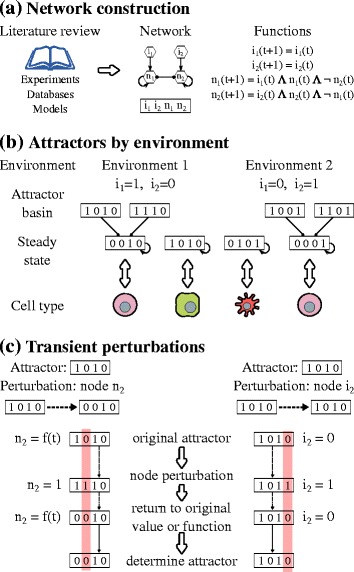
Experimental design of simulations. **a** The network and regulatory functions were grounded on published experimental results. **b** The different inflammatory conditions were simulated by fixing the values of the input nodes of the network that represent the extrinsic cytokines present in the microenvironment. For each simulated condition, the attractors and basins of attraction of the network were obtained. **c** The attractors of the network were perturbed by fixing the value of the target node for one time step and then returning the node to its original function or value; the system attractor was determined

The state of the network *X* can be represented by a vector, that specifies the value of all the nodes of the system. The state of the network will change over time depending on the Boolean functions associated with each node. When the values of a state vector *X* at time *t* are the same as those at time *t + τ*, the system has reached an attractor X* if: X*(t) = X*(t + τ), τ > = 1. An attractor can be interpreted as a stable expression phenotype that correlates or defines a particular cell type [44]. All the states that eventually converge to a solution X* constitute the basin of attraction of such an attractor. We determined the stable states and basins of attraction of the network [Fig. 1] using GINSIM [43] and BoolNet [45]. In all cases synchronous updating was used. Attractors were labelled depending on the expression of both the master transcription factors and cytokines. Labelling was automatized using BoolNetPerturb [46].

#### Effect of the microenvironment

The differentiation of CD4+ T cells depends on the extrinsic cytokines in their microenvironment, which are modeled as inputs in the network. First, we determined a set of biologically relevant microenvironments. For each microenvironment we set the values of the inputs according to the values of the cytokines present or absent in it [Table 1]. Then, we obtained the attractors and their basins and labelled them into different cell types using BoolNetPerturb [46] and the labelling rules for the system [Additional file 1: Table S3].

**Table 1:** Environments of the CD4+ T cell regulatory network

Environment	Cytokines	Active nodes
pro-Th0	no cytokines	None
pro-Th1	IFNγ, IL-12	IFNGe, IL12e
pro-Th2	IL-2, IL-4	IL2e, IL4e
pro-Th17	IL-21 (or IL-6), TGFβ	IL21e, TGFBe
pro-iTreg	IL-2, TGFβ	IL2e, TGFBe
pro-Tr1	IL-10, 1 L-27	IL10e, IL27e

#### Perturbation experiments

To study the plasticity in response to perturbations we used BoolNetPerturb [46]. For every attractor, we perturbed the value of each node for a time step. We then set the function of the node to the bit flipped original value. For example, if a node *i* had an original value of *0*, we set the node’s function from to *f*_*i*_ *= 1*. Then we synchronously evaluated the network for a time step using the modified function [Fig. 1c]. As the perturbation was transient, after a time step the node returns to its original function or, in the case of the inputs, to its original value. Next, we determined the attractor of the perturbed system using synchronous updating and the original function -or value in the case of an input- of the node. If after the perturbation the network returned to an attractor with the same label as the original attractor we established that it was stable to that specific perturbation. If the network arrived to an attractor with a different label, we considered this like a transition from one cell type to another one in response to that perturbation. To verify that the effects of the perturbations were not affected by the simplification of the network we compared a subset of the perturbation experiments in both the minimal and the extended network. We took equivalent attractors for each cell type, then we randomly selected one of the nodes and perturbed it in both networks. Finally we verified that both networks arrived to attractors that could be classified as the same cell type as in the original network without perturbations.

## Results

### CD4+ T cell regulatory network

We expanded the previously published CD4+ T cell transcriptional-signalling regulatory network [13] to include the effect of insulin in the differentiation of CD4+ T cells, according to experimental data [15]. In this network we included multiple molecules like transcription factors, STAT proteins, cytokine receptors, SOCS proteins, and cytokines, among others. We only included direct interactions that have been experimentally validated [Additional file 2: File S1]. Then, we simplified the network [Additional file 2: File S2] to determine the minimal regulatory network that underlies CD4+ T cell fate attainment.

The CD4+ T cell differentiation/plasticity network focused on activated CD4+ T cells in VAT, and was grounded on experimental data [Additional file 2: File S1]. Using this model we studied the role of the different network components in the cellular dynamics and the impact of the environment in cell fate attainment and plasticity patterns [Fig. 1]. The model focuses on activated CD4+ T cells; it assumes that the T cell receptor (TCR) and its cofactors are active. Furthermore, as the model is a minimal network, various components of the system were simplified, but previous simulations guarantee that the main dynamic regulatory motifs and feedback are considered [13]. The extended network can be found as Additional file 3: Fig. S2, and the list of simplified components is available in Additional file 1: Table S1. Given the available data, the model focuses on the observed behaviors in the VAT ignoring the contributions of other tissues. It also focuses on the first stage of hyperinsulinemia, ignoring long- term effects, such as those presented under insulin resistance [10, 38, 40]. The model does not include the dynamic interaction with adipocytes or macrophages, nor the effect hormones like leptin, adiponectin, or sexual hormones, and ignores the differences in glycolysis and lipid oxidation metabolism between effector and regulatory T cells [16–18, 47, 48]. All of the latter may be considered in further explorations.

**Fig. 2:**
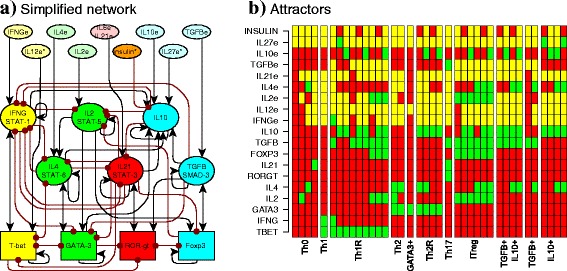
CD4+ T cell regulatory network. **a** The network includes transcription factors (rectangles), intrinsic cytokines and their signaling pathways (ellipses), and extrinsic cytokines and insulin (ellipses). Node colors correspond to cell types in which each molecule is generally expressed (state = 1): Th1 (*yellow*), Th2 (*green*), Th17 (*red*), iTreg (*blue*), and insulin (*orange*). Activations between elements are represented with *black arrows*, and inhibitions with *red dotted arrows*. An * is used to indicate the new nodes considered in this network model with respect to that in [13]. **b** Attractors of the CD4+ T cell regulatory network. Each column corresponds to an attractor. Each node can be active (*green*) or inactive (*red*), extrinsic cytokines may be active or inactive (*yellow*). The following attractors were found in the network: Th0, Th1, Th1R, Th2, GATA3+, Th17, iTreg, TGFβ + IL10+, TGFβ + and IL10+ regulatory cells. Attractors where labeled according to the active transcription factors and intrinsic cytokines

The nodes of the minimal network correspond to transcription factors, signalling pathways and cytokines, while the edges correspond to the regulatory interactions between the nodes and are modelled as Boolean functions [Fig. 1a; Additional file 1: Table S2]. The resulting network contains 19 nodes and 54 interactions [Fig. 2, BioModelsDatabase: MODEL1606020000]. The nodes include: transcription factors (Tbet, GATA3, RORγt, Foxp3), the effector and regulatory cytokines produced by the cell and their signaling pathways (intrinsic) (IFNγ, IL-2, IL-4, IL-21, TGFβ and IL-10), and the cytokines produced by the rest of the immune system (extrinsic) (IFNγe, IL-2e, IL-4e, IL-10e, IL-12e, IL-21e, IL-27e, and TGFβe). To simulate the effect of hyperinsulinemia we extended the previous network to add the regulation of IL-10 by insulin via the AKT pathway [15]; and the STAT3-signaling cytokines: IL-10, IL-6, and IL-21 all use STAT3. We assumed that different pathways mediate IL-10 and IL-6/IL-21 signalling. As the model focuses on activated CD4+ T cells, we assume that the TCR signalling pathway is constitutively active and did not include explicitly this component in the network.

The state of a node represents whether the biological component is active (1) or inactive (0). A node is active when it is capable of altering the regulation of other components of the immune system. For example, CD4+ T cells require a basal level of insulin to survive and we considered this basal level to have a value of 0. A higher insulin concentration, that is capable of affecting IL-10, was set to 1 [15, 49]. In other words, in our model, hyperinsulinemia is simulated by setting the “insulin” node to 1. We are aware that this is an oversimplification of the relationship between our network and insulin, and it does not really consider the feedback between both. Nonetheless, the simulation we propose here is useful to explore the dynamics of the system and how high insulin levels alter the stability of different CD4+ T cells.

Cytokines can be produced by the cell (intrinsic) or by other cells of the immune system (extrinsic). Such extrinsic cytokines constitute the microenvironment and have an important role in CD4+ T cell differentiation and plasticity. Extrinsic cytokines were considered as inputs of the system [Fig. 1b]. To study the effect of the microenvironment we focused on six biologically relevant environments: pro-Th0 or resting, pro-Th1, pro-Th2, pro-Th17, pro-iTreg, and pro-Tr1 [Table 1].

The stable states to which a regulatory network converges are called attractors, and can be interpreted as the expression profiles of the considered components that are characteristic of different cell types [50, 51] [Fig. 1b]. We labelled each attractor according to the active transcription factors and intrinsic cytokines [Additional file 1: Table S3]. Th0, resting T cells, were defined as expressing no transcription factors or regulatory cytokines. Th1 was defined as having Tbet and IFNγ active, Th2 as GATA3 and IL-4 active and GATA3+ (a Th2-like cell type) as GATA3 + IL4-. Th17 cells are characterized by the expression of RORγt and STAT3 signalling mediated by IL-6 or IL-21, all of which require the presence of TGF-βe. The iTreg type has Foxp3 and TGFβ, IL-10 or both, all of which require the presence of IL-2e. T regulatory Foxp3-independent cells feature IL-10 (Tr1), TGF-β (TGFβ+) or both (IL10 + TGFβ+), without expressing Foxp3. Th1 regulatory cells (Th1R) express a regulatory cytokine and T-bet [24, 25]. Th2 regulatory cells (Th2rR) express a regulatory cytokine and GATA3. The attractors obtained by the CD4 + T cell network correspond to configurations that are characteristic of: Th0, Th1, Th1R, Th2, GATA3+, Th2R, Th17, iTreg, Tr1, TGFβ + and TGFβ + IL10+ CD4+ T cells [Additional file 1: Fig. S1] [20, 15].

### Effect of insulin on CD4+ T cell differentiation

**Fig. 3:**
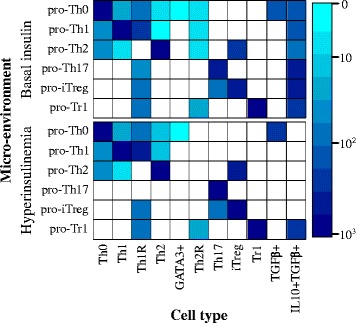
Effect of the microenvironment on CD4+ T cell differentiation. The values of the extrinsic signals of the TSRN were fixed according to different polarizing microenvironments. The color corresponds to the size of the basins of attraction on a logarithmic scale. No color means the cell type is not present in the polarizing microenvironment

To simulate the effects of insulin, we obtained the attractors in the different microenvironments under both, basal (state of the “insulin” node to 0) or high levels (state of the “insulin” node to 1) of insulin [Fig. 3]. To simulate the different environments we fixed the values of the input nodes according to each environment as listed in Table 1. Then, we determined and labelled the resulting attractors to obtain the predicted cell types under each environment and insulin condition [Fig. 1b].

Our model shows that in effector polarizing environments with basal levels of insulin, like pro-Th1, pro-Th2 and pro-Th17, effector and regulatory cells coexist. Restive cells, labelled as Th0, can coexist in pro-Th0, pro-Th1 and pro-Th2 environments. In a pro-iTreg environment there is a coexistence of iTreg and Th17 cells. But in a pro-Tr1 we see a strong polarization towards regulatory T cells and no effector CD4+ T cells. We observed that in the presence of high levels of insulin there is a marked decrease of the attractors that express IL-10 (Th1R, Th2R, and IL10 + TGFβ+), and the remaining attractors tend to express TGFβ. There is an increase in the size of the attraction basins of the Th17 and Th1 attractors. This is particularly notable in the pro-Th17 insulin environment, where the Th1R and IL10 + TGFβ + disappear, and the network converges to Th17. In the case of the Th1 attractor the increase in its basin size is smaller. Interestingly, this behavior corresponds to the observed increase in Th1 and Th17 and the decrease in Treg cells and IL-10 under obesity-associated chronic inflammation. The only exception to this pattern was observed under the pro-Tr1 environment that remains unchanged by the level of insulin.

### Effect of insulin on CD4+ T cell plasticity

CD4+ T cells are plastic and dynamically change from one type to others, depending on the microenvironment and transient perturbations or initial conditions. This implies that these cells configurations and behavior can be altered dynamically. The multi-stable Boolean network model used here is a useful tool to study CD4+ T cell plasticity [13] under basal and high insulin levels. To explore this, we transiently perturbed the attractors [Table 1] under the constitutive presence of basal (0) and high levels of insulin (1). For each attractor, we fixed the function of the target node to the bit flipped original value, and then we synchronously evaluated the network for a time step using the fixed function [Fig. 1c]. Then, we returned the node to its original function, and determined the resulting attractor [Fig. 1c]. We established that a labelled attractor was stable to a perturbation if it returned to the same cell type after the transient perturbation. When the system transitioned to an attractor that corresponds to a different cell type, we considered the original attractor to be plastic under transient perturbations.

**Fig. 4:**
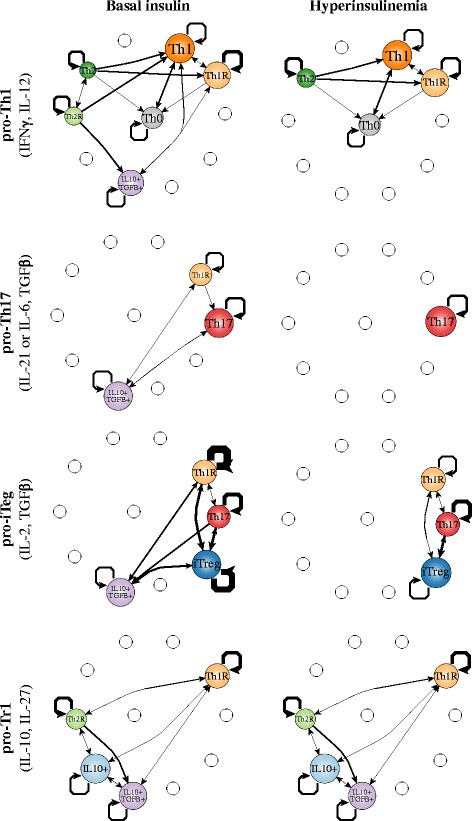
Cell fate map under different microenvironments. The values of the extrinsic signals of the CD4+ T regulatory network were fixed according to different polarizing microenvironments as listed in Table 1, and the resulting attractors were transiently perturbed for one time step. Nodes correspond to cell types, node size is proportional to the number of configurations in a basin of attraction. Edges represent transitions from one cell type to another, their width represents the number of times the transition occurred, self-loops correspond to perturbations in which the network returned to the original cell type. The following microenvironments were studied: pro-Th1, pro-Th1+ Insulin, pro-Th17, pro-Th17 + Insulin, pro-iTreg, pro-iTreg + Insulin, pro-Tr1 and pro-Tr1 + Insulin

Our results suggest that the effect of insulin on the differentiation and plasticity of CD4+ T cells depends on the cytokines that are present in the microenvironment [Fig. 4, Additional file 4: File S3]. In each microenvironment, without insulin, most of the transitions lead the system to the favored cell type, which tends to be the most stable one, as expected. But in these cases, other cell types also coexist in the environment, especially regulatory cell types, even though the attractors that characterize them are less stable. Under high levels of insulin, that simulates an acute hyperinsulinemia condition, the CD4+ T plasticity patterns are altered. In general, the activation of insulin: (1) causes the loss of the regulatory attractors, particularly Tr1, reduces cell stability, and the number of transitions towards the original cell type. This is particularly notable in the pro-Th17 environment, where Th17 is the only possible attractor. In the case of the pro-iTreg environment, there is a coexistence of iTreg with Th17. This is caused by the extrinsic TGFβ. The role of TGFβ is bivalent, as it can induce both regulation and inflammation through iTreg and Th17 cells. In the case of TGFβ, insulin shifts the equilibrium towards inflammatory cell types. The addition of insulin caused the loss of the IL10 + TGFβ + attractor, stabilized Th17, and reduced the stability of iTreg and Th1R. In the only case that this did not occur, was under the pro-Tr1 environment, where a regulatory phenotype is attained independently of the insulin level, avoiding a pro-inflammatory condition even under the presence of hyperinsulinemia.

### The role of IL-10 on CD4+ T cell plasticity alterations under normal and hyperinsulinemic conditions

**Fig. 5:**
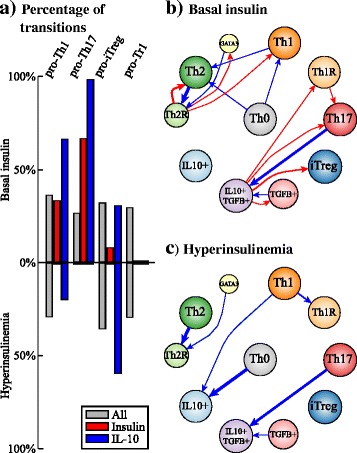
Transitions between cell types caused by the transient activation of the insulin and the IL-10 nodes. **a** Percentage of transitions between attractors in response to transient perturbations of: all nodes (*grey*); insulin (*red*) or IL-10 nodes (*blue*) under basal and high levels of insulin. **b**, **c** Transitions between cell types caused by the transient activation of: insulin (*red*) or IL-10 nodes (*blue*) under basal (**b**) and high levels (**c**) of insulin

We assessed how many transitions among attractors were caused by transient perturbations of insulin and IL-10 under normal and hyperinsulinemic conditions [Fig. 5]. On average, perturbations of any node caused transitions to new cell types in 38% of the cases. But the number of transitions between cell types varied according to the node and the microenvironment. The two nodes whose transitions in response to transient perturbations varied more among environments (i.e., basal insulin versus hyperinsulinemia) were IL10 and, trivially, INSULIN itself [Additional file 4: File S3]. The transient increase of insulin caused transitions towards inflammatory or TGFβ producing cell types under basal insulin level, while, as expected, under hyperinsulinemia the transient activation of insulin did not cause any further transitions. The attractors of the pro-Th17 environment with basal levels of insulin were very sensitive to perturbations in the insulin node, while the attractors found in the pro-iTreg and the pro-Tr1 environment were robust to this perturbations. The transient activation of IL-10 caused transitions towards regulatory cell types. The attractors of the pro-Th1, pro-Th17 with basal levels of insulin and the pro-iTreg with high level of insulin environments were, on the other hand, very sensitive to transient perturbations of IL-10. In environments with basal levels of insulin, the transient activation of IL-10 caused some transitions towards Th1 and Th2 cell types in pro-Th1 and pro-Th2 environments, respectively. In these cases, the transient activation of IL-10 was sufficient to destabilize the attractor but not to shift the network towards a regulatory cell type.

## Discussion

The theoretical simulation study presented here suggests that the impact of hyperinsulinemia on the inflammatory response [15], is mediated by the multi-stable dynamic GRN in [13]. The dynamical model and simulations provide a system-level analytical framework underlying observed cell behaviors and offer predictions that will require experimental validation [Table 2]. Overall, our simulation study provides a platform to explain the relationship between hyperinsulinemia and altered stability and proportions of T regulatory cells that have been observed in adipose tissue [3, 36–38, 40]. It also highlights and provides a dynamic explanation for the different roles of TGFβ and IL-10 [30–33].

**Table 2:** Observations and predictions of the model

Predictions	
Environment	Prediction
	The cell type favored by the environment is more stable than the coexisting cell types
Insulin high	Decrease in Tr1 stability
Insulin high	Increase in Th17 stability
Pro-Th17(TGFBe, IL21e) + insulin high	Only the Th17 attractor is attainable
Pro-Tr1 (IL10e, IL27e)	Tr1, IL10 + TFGB + Foxp3-, Th1R and Th2R cells can coexist independently of the level of insulin
Observations	
Environment	Observation
	Effector and regulatory cells can coexist in a microenvironment [29].
Pro-Treg (IL2e, TGFBe)	Tregs, Th17, IL10 + TFGB + Foxp3- and Th1R cells can coexist [29].
Insulin high	Decrease in the number of Tr1 cells [15].
Insulin high	TGFβ-producing cells are unaltered [15].
Insulin high	Th1 cells basin of attraction increases slightly [3, 36–38].
Insulin high	Th17 cells basin of attraction clearly increases [3, 36–38].

The model shows that in pro-Th1, pro-Th2, pro-Th17 and pro-iTreg microenvironments, effector and regulatory cells coexist. This pattern is observed in any disease, where it is common to find cells from different subsets, even if a specific one is over-represented [52]. Moreover, the simulations predict that various types of regulatory cells will predominate depending on the environment, being especially important to distinguish Foxp3+ and Foxp3- regulatory T cells. Future experiments should consider that CD4+ T cells are highly heterogeneous, phenotypically plastic and sensitive to the microenvironment. A CD4+ T cell can express markers for more than one cell type at the same time, and its expression patterns can change over time. This is the case especially for regulatory T cells.

Our model simulations suggest that future experimental studies should pursue evaluations of the levels of Foxp3, IL-10, and TGFβ expression levels to systemically distinguish Treg (Foxp3 + CD25high), Tr1 (Foxp3-IL10+), Th3 (Foxp3-TGFβ+) and Th1R and Th2R hybrid cell types. Distinguishing these cell types will be necessary to understand the different roles that they play in obesity-associated chronic inflammation. Future assays should also consider multiple transcription factors and cytokines, carefully separate CD4+ T cell populations and compare their behaviors in different tissues.

Our simulation results recovered altered stabilities of different CD4+ T cells in a way that is qualitatively similar to the alterations reported for CD4+ T cell populations in murine models and humans during obesity-associated chronic inflammation [3, 36–38, 40]. In the presence of hyperinsulinemia, increased proportions of Th1 and Th17 cells and decreased proportions of regulatory T cells have been observed [3, 37, 38]. In our model and specifically in a pro-Th17 environment, the presence of insulin predicts a complete shift towards Th17 cells. In contrast, in a pro-Th1 environment the Th1 attractor alteration is less dramatic than the alterations observed in vivo, probably because of the involvement of macrophages in the real condition, that are not considered in the simulation model of this study [3, 37, 38].

The model also provides an explanation to some apparently paradoxical behaviors observed in CD4+ T regulatory cell populations during obesity-associated chronic inflammation. TGFβ can promote both inflammatory Th17 cells and regulatory Tregs, and transitions between both subsets have been observed [3, 37, 38, 53, 54]. The model presented here provides a mechanistic and systemic explanation to the fact that Th17 cells and iTregs are closely related and that Th17 cells can be observed sometimes during the iTreg response. TGFβ is necessary for the differentiation of both subsets, and our model predicts that transient signaling via the STAT3 pathway may be enough to shift some cells towards Th17. In obesity, Tregs expression profiles are similar to inflammatory T cells [36]. Transfer and depletion of adipose Treg cells have been reported to both, improve or worsen insulin sensitivity, depending on the model and the population studied [37, 39, 40]. Such apparently paradoxical behaviors can be explained by the relationship between TGFβ and IL-10 in the context of the dynamic regulatory network model used here. Under hyperinsulinemia, Th17 cells become more stable while Tr1 cells are lost. The remaining regulatory cells express TGFβ that is involved in Th17 differentiation, while insulin alters iTregs stability. In this way, the model predicts that under hyperinsulinemic inflammatory environments, especially under pro-Th17 conditions, T regulatory cells are lost and the rest become unstable. In contrast, a pro-Tr1 environment can induce regulatory T cells, regardless of the level of insulin in the environment. Nonetheless, while this pro-regulatory environment might decrease inflammation, it may have adverse effects because inflammation is relevant for the function of adipose tissue [55].

The model presented here also predicts that the transitions between cell types vary depending on the microenvironment and the perturbed node. Transient activation of insulin is sufficient to cause transitions towards inflammatory or TGFβ + cells, while transient activation of IL-10 is sufficient to cause transitions towards regulatory cells. The over-activation of the Akt/mTOR pathway by hyperinsulinemia plays a key role in this shift [15]. This pathway has been reported to be critical for cell fate determination in response to the TCR signalling [21]. It is highly probable that CD4+ T cell differentiation relies in redundant modules that together contribute to the dynamical robustness of the system [9, 56]. The stability of the different cell types will also vary depending on the microenvironment and the perturbation. We predict that the cells in a pro-Th17 environment are more sensitive to transient increases in insulin, while the cells in a pro-iTreg and pro-Tr1 environments are more stable under the same perturbation.

The model used here considers a minimum regulatory network underlying CD4+ T cell differentiation and plasticity under hyperinsulinemia, and as such is useful as a starting point for a systemic integration of the complex network underlying CD4+ T cell differentiation and plasticity under basal and increased insulin levels. In order to address more realistic conditions, however, the model should encompass additional components in order to study the dynamics of additional cells and signals that are fundamental to fully understand obesity-associated chronic inflammation, as well as to fully consider the feedback between the CD4+ T regulatory module and insulin metabolism. For example, since the network used here is a minimal model, it ignores cytokines such as IL-1 and TNFα, the role of hormones produced by adipocytes like leptin, adiponectin, or sexual hormones. Furthermore, additional cell types like adipocytes and macrophages that play important roles during obesity-associated chronic inflammation could be considered as well in future simulations [16–18, 47, 48]. Leptin regulates appetite in the long term, stimulates energy expenditure and decreases insulin secretion. Obese persons have high blood leptin levels, but they also show leptin resistance and hence, because insulin secretion is not well regulated either, with time can develop type II diabetes. Nonetheless, adiponectin sensitizes tissues to insulin action and has protective effects on beta cells, but their plasma level decreases in obese people. For this reasons it is reasonable not to take them into account in this model.

Cell metabolism might also provide useful insights for the differences and relationships among effector, resting and regulatory CD4+ T cells [14]. For now, we used the model to focus on assessing the role of hyperinsulinemia on the differentiation dynamics of an activated CD4+ T cell in VAT [15]. Future studies will incorporate the TCR and insulin signaling pathways and the contrasting metabolism that has been documented among effector, resting and regulatory conditions.

Furthermore, future efforts should become more quantitative and enable considerations of the strength and length of the signals in the dynamics of the immune system. Continuous, asynchronous or signal length studies may be useful to assess different metabolic disorders and chronic inflammation illnesses, as well as the actual timing and progression of the obese inflammatory response, or the treatment of these health conditions [21].

Also, the model used here, still simplifies the microenvironment, that is much more complex in vivo. For example, it is interesting to assess how the length and intensity of the small initial signals that occur in response to nutrient overload, eventually give rise to significant alterations associated to obesity-associated chronic inflammation [16]. Further studies of the effect of transient signals in an asynchronous or continuous version of the minimal and extended CD4+ T cell regulatory network, will likely yield important insights concerning such temporal patterns.

Concerning the simplification of the model, a formal approach was considered to conserve the dynamic properties of the network. Furthermore, the effect of some perturbations in a subset of the attractors was simulated and compared in both the extended and the minimal network to warrant that the observed behavior was not an artifact of the simplification. Future analyses should systematically analyze the minimal system while verifying that critical perturbations behave in the same way in the extended versions, evaluating the role that the simplification and the updating scheme used in the present model could have on the transient behaviors observed in vivo. For example, delays in signalling pathways can affect the heterogeneity, plasticity, and population dynamics of CD4+ T cells [21]. The simplification of the model may also hide some of the molecules involved in the system, complicating the experimental validation or hindering some alternative therapeutic approaches.

## Conclusions

The model presented here combines the regulatory network that underlies CD4+ T cell attainment with the effect of hyperinsulinemia to provide a mechanistic explanation to the dynamical behavior of CD4+ T cells during obesity-associated chronic inflammation. High insulin levels affect the differentiation and plasticity of CD4+ T cells, favoring inflammatory, resting or TGFβ + producing cell types and reducing the stability of regulatory cell types. Despite the limitations of the model, the methodological framework proposed here is useful for studying the relationship between CD4+ T cells differentiation and plasticity dynamics and hyperinsulinemia. The framework put forward here may be extended and used to understand how other pathways, modules, and systems interact with the regulatory module of this study, to further uncover and explore system-level behaviors. The approach proposed here may also be useful for toxicological studies, and for providing predictions concerning the biological impact of drugs, assessing therapeutic targets or secondary effects.

## Additional files

Additional file 1: Figure S1. Extended CD4+ T cell regulatory network. **Table S1.** Node simplification of the CD4+ T cell regulatory network. **Table S2.** Rules of the CD4+ T cell regulatory network. **Table S3.** Labeling rules of the CD4+ T cell regulatory network. (DOC 315 kb)

Additional file 2: File S1. References of the CD4+ T cell regulatory network. **File S2.** Simplification of the CD4+ T cell regulatory network. (XLS 40 kb)

Additional file 3: Network simplification. **Figure S2.** Attractors of the CD4+ T cell regulatory network. (DOC 187 kb)

Additional file 4: File S3. Perturbation tables of the CD4+ T cell regulatory network. (XLS 243 kb)
