# Supplementary material for: The CD4+ T cell regulatory network mediates inflammatory responses during acute hyperinsulinemia: a simulation study
Source: BMC Syst Biol. 2017 Jun 26;11:64. doi: 10.1186/s12918-017-0436-y (PMC5485658; doi:10.1186/s12918-017-0436-y)
Supplement: Supplementary file 1 — Extended CD4+ T cell regulatory network. Table S1. Node simplification of the CD4+ T cell regulatory network. Table S2. Rules of the CD4+ T cell regulatory network. Table S3. Labeling rules of the CD4+ T cell regulatory network. (DOC 315 kb) [file 12918_2017_436_MOESM1_ESM.doc]

Supplementary Material

**The CD4+ T cell regulatory network mediates inflammatory responses during acute hyperinsulinemia: a simulation study**

M.E. Martinez-Sanchez, M. Hiriart, E. R. Alvarez-Buylla*

*** Correspondence:** Corresponding Author: [eabuylla@gmail.com](mailto:eabuylla@gmail.com)

# Supplementary Data

**Supplementary File 1: References of the CD4+ T cell regulatory network**. <ThInsulin-References.xls>

**Supplementary File 2: Simplification of the CD4+ T cell regulatory network**. <ThInsulin-Transitions.xls>

**Supplementary File 3: Perturbation tables of the CD4+ T cell regulatory network**. <ThInsulin-Transitions.xls>

# Supplementary Figures and Tables

## Supplementary Figures


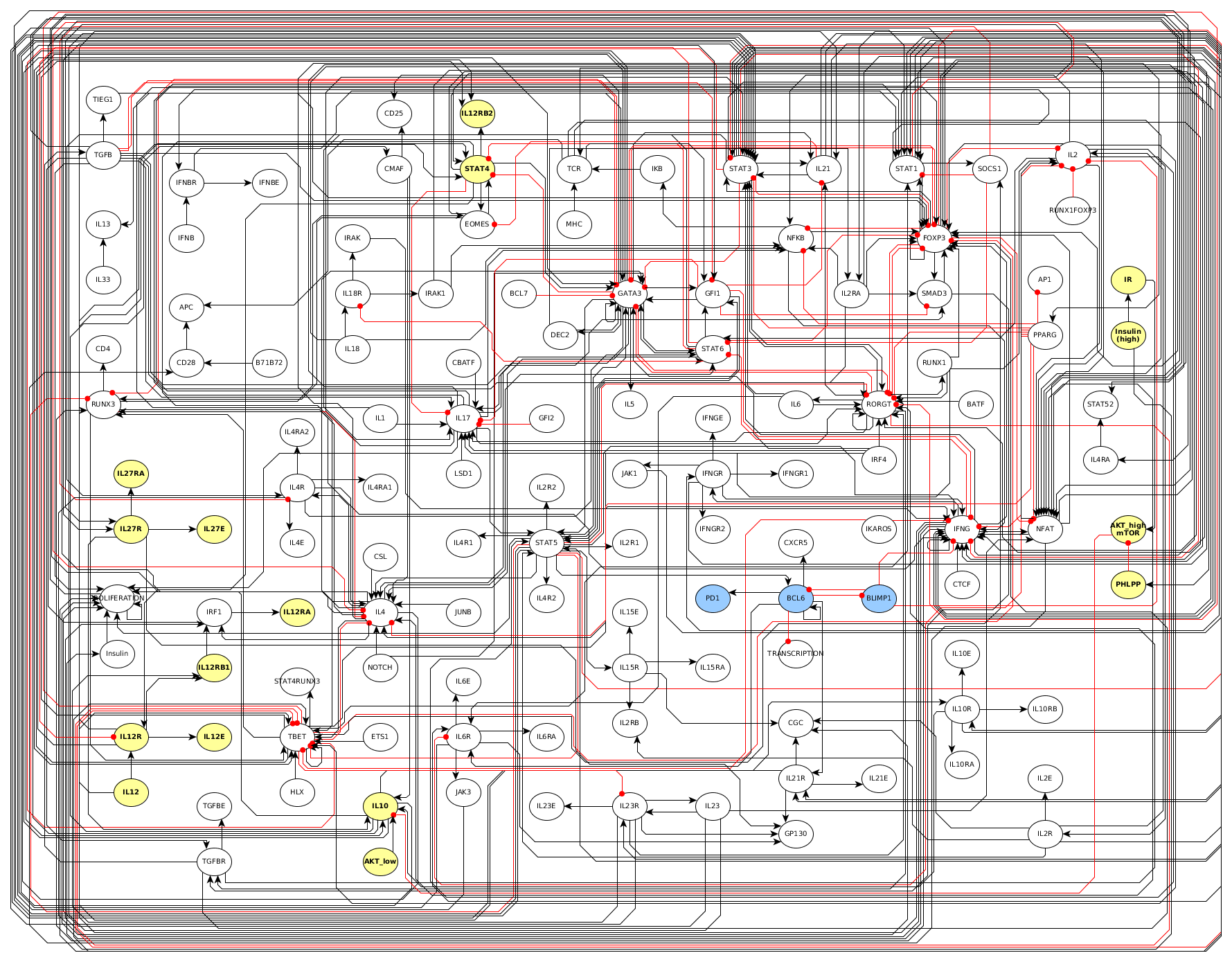


**Supplementary Figure 1: Extended CD4+ T cell regulatory network**. The CD4+ T cell regulatory network includes transcription factors, intrinsic cytokines and their signaling pathways (including STAT and SOCS proteins), and extrinsic cytokines and insulin, among others. Yellow nodes correspond to molecules added in this paper with respect to previous network [13], and blue nodes correspond to molecules removed for this paper with respect to those in [13]. The interactions proposed in the network between elements are grounded on experimentaly verified interactions. Activations between elements are represented with black arrows, and inhibitions with red dotted arrows. The network was then simplified using methods that maintain the dynamics of the system (see Supplementary material).

## Supplementary Tables

**Supplementary Table 1. Node simplification of the CD4+ T cell regulatory network**.

| **Node** | **Biological molecules** |
| --- | --- |
| TBET | T-bet, Eomes, Runx3, Ets1, Hlx, RELB |
| IFNG | IFNγ, IFNγR, STAT1 |
| GATA3 | GATA3, Dec2, Fli1, Gfi1, Irf4, TCF1, Ikaros |
| IL2 | IL-2, IL2-R(α,β,γ), STAT5 |
| IL4 | IL-4, IL-4R, STAT6 |
| RORGT | RORγt, Irf4, BATF, Runx1, c-REL, EBF1 |
| IL21 | IL-6, IL-6R, IL-21, IL-21R, IL-23, IL-23R, STAT3 |
| FOXP3 | Foxp3, Et1, FOXO1, CREB, c-Rel, opera, PHLPP |
| TGFB | TGFβ, TGFβR, SMAD3, TGFβ signaling pathway, TIEG1 |
| IL10 | IL-10, IL-10R, IL10 signaling pathway, c-maf, CREB, IRF1, SP1 |
| IL12e | IL-12, IL-12R, IRAK1 |
| IL21e | Extrisic IL-6, IL-21, and IL-23 |
| TCR | AP-1, ICOS, NFAT, NFkB, CD28, Jun, etc |
| Insulin -| IL-10 | Akt (high), mTOR |
| IL10 -| IFNG | SOCS3 |
| IL4 -| IFNG | SOCS3 |
| TGFB -| IFNG | SOCS3 |
| STAT3 -| IL2 | SOCS1 |
| IL10 -| IL2 | SOCS2 |
| IFNG -| IL2 | SOCS1, SOCS2 |
| STAT3 -| IL4 | SOCS1 |
| IFNG -| IL4 | SOCS1 |
| IL10 -| STAT3 | SOCS3 |
| IL4 -| STAT3 | SOCS3 |
| IFNG -| STAT3 | SOCS3 |

**Supplementary Table 2. Rules of the CD4+ T cell regulatory network**.

| Node | Function |
| --- | --- |
| TBET | ((IFNG | (IL12e & ! (IL21 | IL4 | IL10)) ) | TBET) & ! (IL4 | GATA3 | IL21) |
| IFNG | (IFNGe | ((IFNG | TBET) & ! (GATA3 | TGFB))) & ! (IL21 | IL4 | IL10) |
| GATA3 | ((IL2 & IL4) | GATA3) & ! (TBET | TGFB | IL21 | IFNG) |
| IL2 | (IL2e | (IL2 & ! FOXP3)) & ! (IFNG | IL21 | (IL10 & ! FOXP3)) |
| IL4 | (IL4e | (GATA3 & (IL2 | IL4) & ! TBET)) & ! (IFNG | IL21) |
| RORGT | (IL21 & TGFB) & ! (TBET | FOXP3 | GATA3) |
| IL21 | (IL21e | IL21 | RORGT) & ! (IFNG | IL4 | IL10 | IL2) |
| FOXP3 | (IL2 & (TGFB | FOXP3)) & ! (IL21 | RORGT) |
| TGFB | TGFBe | ((TGFB | FOXP3) & ! IL21 ) |
| IL10 | IL10e | (IL10 & (IFNG | IL21 | TGFB | GATA3 | IL27e) & ! INSULIN) |
| IFNGe | IFNGe |
| IL12e | IL12e |
| IL2e | IL2e |
| IL4e | IL4e |
| TGFBe | TGFBe |
| IL10e | IL10e |
| IL27e | IL27e |

**Supplementary Table 3. Labeling rules of the CD4+ T cell regulatory network**.

| Labels | Rules |
| --- | --- |
| Th0 | ! (TBET | GATA3 | RORGT | FOXP3 | IL10 | TGFB) |
| Th1 | (TBET & IFNG) & ! (IL10 | TGFB | FOXP3) |
| TBET+ | TBET & ! (IFNG | IL10 | TGFB | FOXP3) |
| Th1R | TBET & (IL10 | TGFB | FOXP3) |
| TH2 | (GATA3 & IL4) & ! (IL10 | TGFB | FOXP3) |
| GATA3+ | GATA3 & ! (IL4 | IL10 | TGFB | FOXP3) |
| Th2R | GATA3 & (IL10 | TGFB | FOXP3) |
| Th17 | RORGT & IL21 & ! IL10 |
| RORGT+ | RORGT & ! (IL21 | IL10) |
| iTreg | FOXP3 & TGFB & ! (TBET | GATA3 | RORGT) |
| IL10+ | IL10 & ! (TBET | GATA3 | FOXP3 | RORGT) |
| TGFB+ | TGFB & ! (TBET | GATA3 | FOXP3 | RORGT) |
