# Supplementary material for: The CD4+ T cell regulatory network mediates inflammatory responses during acute hyperinsulinemia: a simulation study
Source: BMC Syst Biol. 2017 Jun 26;11:64. doi: 10.1186/s12918-017-0436-y (PMC5485658; doi:10.1186/s12918-017-0436-y)

**Supplementary Material**

**Network Simplification**

Mariana Martinez-Sanchez, Marcia Hiriart, Elena R. Álvarez-Buylla

The differentiation and plasticity of CD4+ T lymphocytes is the result of the concerted action of many components (cytokines, receptors, transcription factors, etc). The large number of components makes it convenient to simplify the resulting network to facilitate the analysis and explore a sufficient set interactions to recover the configurations of the molecular components, that have been identified as important during CD4+ T cell fate attainment.

**Network construction**

We considered that a node is active if there is enough amount of protein or mRNA to be functional and affect the differentiation of CD4+ T lymphocytes. A transcription factor is active if it is present in enough quantity and in a conformation that can alter the expression of its target genes. A transcription factor or cytokine is active if it is present in enough amount and in a conformation that can form a functional complex with its receptor. A receptor is active if it forms a complex that can activate its downstream signaling components. A STAT protein is active if it is phosphorylated and forms a dimer capable of translocating to the nucleus and affecting the expression of its target genes.

A protein or gene may be expressed at a basal level, but does not necessarily affect the differentiation of the cell at that level of expression. For example, insulin is necessary for T cell survival and proliferation. In the absence of insulin effector T cells may enter apoptosis. However, a high level of insulin can inhibit IL-10 expression. For example, according to [15].

| **Expression** | **Concentration** | **Phenotype** | **Value** |
| --- | --- | --- | --- |
| No insulin | 0 | Cell death | - |
| Basal insulin | 0.002 ng/ml | Survival | 0 |
| Hyperinsulinemia | 10-100 ng/ml | Inhibition IL-10 | 1 |

We consider that a node is active when it can alter the expression of its downstream targets. For example, IL-10 requires low levels of Akt (activated by the TCR complex or insulin) to maintain its expression, but high concentrations of insulin inhibit IL-10 by overactivating the AKT/mTOR pathway. Given that one of the assumptions of the model is that the TCR, its cofactors, and downstream signals are present in enough amounts to enable the activation and expression of the cytokines and transcription factors, we can simplify the expression of Akt and its effect over IL-10 as an inhibition function.

| **Akt concentration** | **AKT(t) value** | **IL10(t+1) value** |
| --- | --- | --- |
| No AKT | - | - |
| Akt low | 0 | 1 |
| Akt high | 1 | 0 |

Weak interactions were ignored in our model. Interactions between genes and proteins are weak when they increase or decrease the expression of a gene or protein but are not necessary or sufficient to cause changes in differentiation. For example, the IL-23 receptor (IL-23R) is part of the maintenance of CD4+ T cells and promotes the phosporilation of STAT3. In turn, STAT3 increases the expression of the IL-23R. In our model, a receptor is active if it forms a complex that can activate its downstream signaling components. This means that, even if there is a low initial concentration of IL-23R, it may be enough to bind to the cytokine, and phosphorilate its downstream signal, STAT3. The result is that in the model IL-23R can induce the phosphorilation of STAT3 (STAT3 = 1), even if the transcription factor affects its expression levels and affinity.

**Boolean Logic Reduction Method**

To simplify the network we employed a Boolean reduction method proposed in Villarreal et al, 2012. For simplicity, we illustrate only the simplification scheme of the interactions between IL-6, IL-21, IL23 and STAT3. The three cytokines bind to their receptors and activate STAT3 through their receptors. IL-21 expression can be induced in CD4+ T cells with the collaboration of STAT3 and RORgt, and is part of the feedback loop between RORgt and IL-21. STAT3 also increases the expression of the IL-23R. However, given that this is a weak interaction, we will ignore it. These interactions can be characterized by a set of logical propositions which satisfy the following mapping:


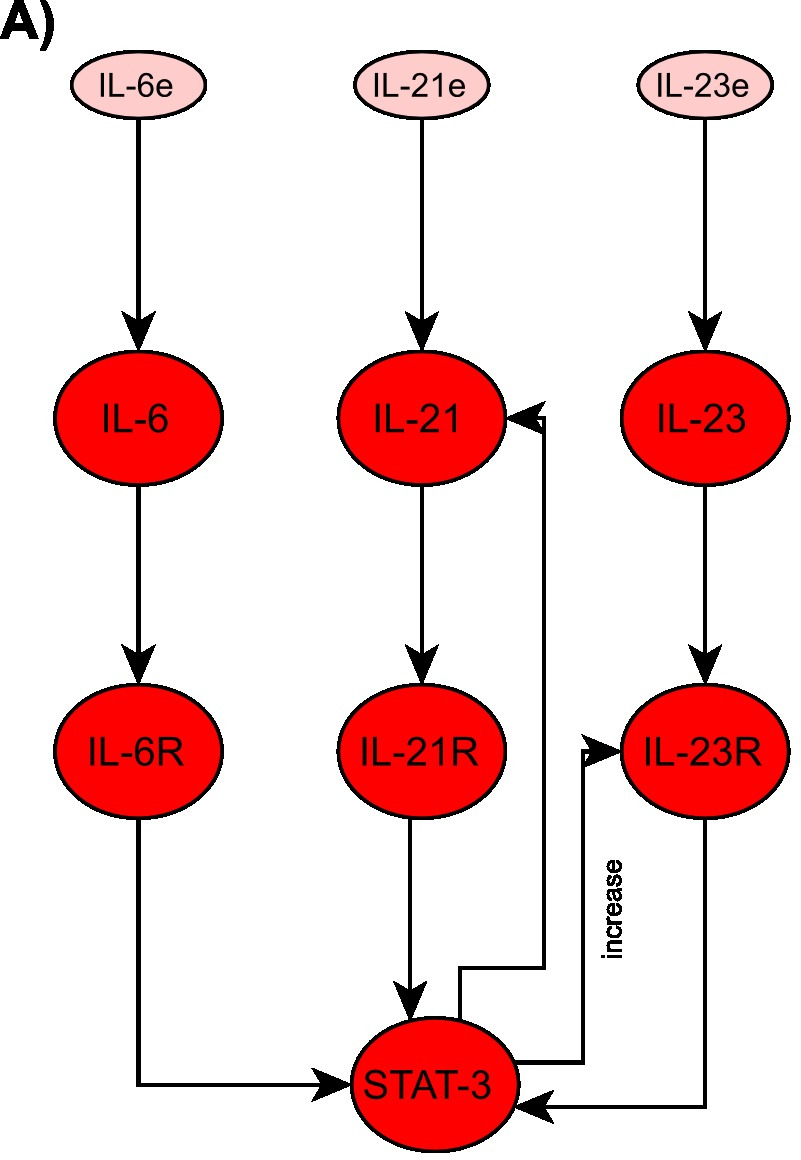


IL6e(t+1) = IL6e(t)

IL6(t+1) = IL6e(t)

IL6R(t+1) = IL6(t)

IL21e(t+1) = IL21e(t)

IL21(t+1) = IL21e(t) or STAT3(t)

IL21R(t+1) = IL21(t)

IL23e(t+1) = IL23e(t)

IL23(t+1) = IL23e(t)

IL23R(t+1) = IL23(t)

STAT3(t+1) = IL6R(t) or IL21R(t) or IL23R(t)

Considering that the expression level of node *N* at a time *t* is represented by *N(t),* the steady states that represent different phenotypes are determined by the condition *N(t+1) = N(t)*. In that case, the mapping becomes a set of coupled Boolean algebraic equations. The explicit expressions of the attractors are then obtained by performing the algebraic operations according to the axiomatic of Boolean algebra (see Villarreal et al, 2012). This results in a set of identities that can be employed to determine the system’s attractors. Thus, the regulatory network attractors are summarized by the expression values of the nodes pertaining to a concise set of Boolean expressions:


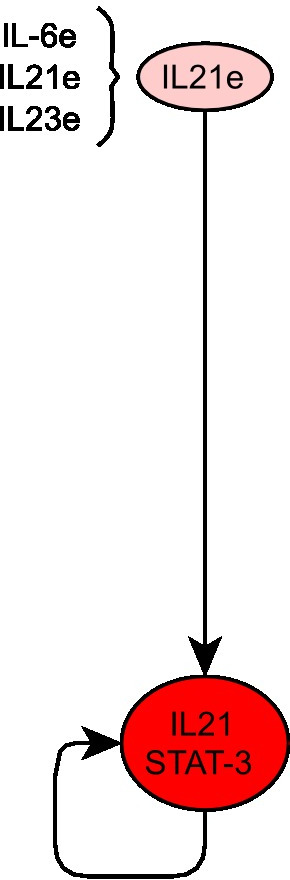


IL6e = IL6e IL21e = IL21e

IL23e = IL23e

STAT3 = IL6e or (IL21e or STAT3) or IL23e

However, given that IL6e, IL21e, and IL23e have the same effect on the STAT3 node we can further simplify the system to obtain the set of Boolean expressions as follows:

IL21e = IL21e

STAT3 = IL21e or STAT3

Finally, we can verify that the attractors of the extended regulatory network correspond to those of the simplified model for the components that are kept in the reduced version.


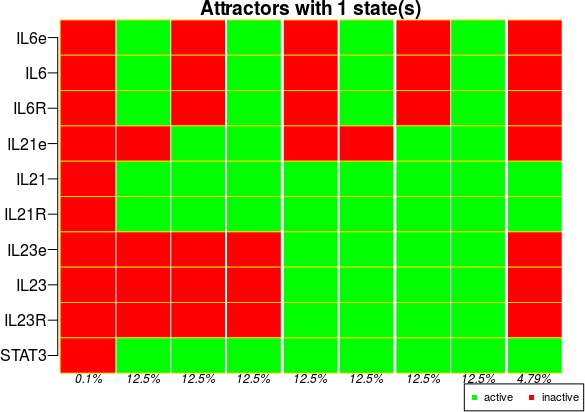

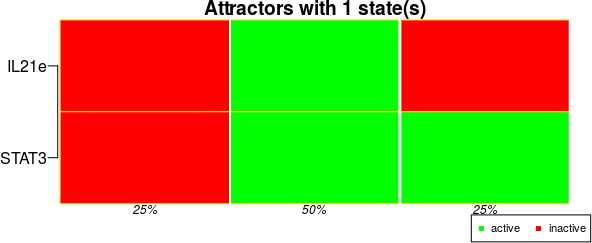

Supplement: Supplementary file 3 — Network simplification. Figure S2. Attractors of the CD4+ T cell regulatory network. (DOC 187 kb) [file 12918_2017_436_MOESM3_ESM.doc]
